# sCLU as prognostic biomarker and therapeutic target in osteosarcoma

**DOI:** 10.1080/21655979.2019.1621136

**Published:** 2019-06-11

**Authors:** Jinfeng Ma, Weiliang Gao, Jisheng Gao

**Affiliations:** a Department of Spine Surgery, The Affiliated Hospital of Qingdao University, Qingdao, Shandong, China; b Department of Spine Surgery, The 107 Hospital of the People’s Liberation Army, Yantai, Shandong, China

**Keywords:** Osteosarcoma, secretory secretory apolipoprotein J/clusterin, chemotherapy, invasion, metastasis, apoptosis

## Abstract

Osteosarcoma (OS) is the most common primary malignant bone tumor. Secretory apolipoprotein J/clusterin (sCLU) is overexpressed in many cancers; however, its role in OS has not been previously investigated. The objectives of this study were to address this question and also to assess the clinical value of sCLU as a prognostic biomarker and therapeutic target by comparing sCLU expression in human OS (n = 106), normal bone (n = 16), fibrous dysplasia (n = 9), and ossifying myositis (n = 11) tissues and by evaluating the effect of sCLU silencing on OS growth, invasion, and chemosensitivity in vitro and in vivo. We found that sCLU was highly expressed in OS tissue specimens, which was positively correlated with metastatic disease and negatively correlated with response to chemotherapy. *sCLU* knockdown in KHOS cells inhibited proliferation and invasion and increased apoptosis as well as sensitivity to the chemotherapy drug gemcitabine (Gem). In a mouse xenograft model, sCLU depletion suppressed lung metastasis and enhanced the effects of Gem, thereby slowing KHOS tumor growth. These results indicate that sCLU overexpression is a biomarker for malignant transformation of OS and that therapeutic strategies targeting sCLU may be an effective treatment for OS.

**Highlights**

● Secretory apolipoprotein J/clusterin (sCLU) is overexpressed in osteosarcoma (OS).

● sCLU overexpression is associated with metastasis and chemoresistance.

● Silencing sCLU inhibits metastasis and enhances chemosensitivity in OS cells.

sCLU is a biomarker for metastatic OS and a potential therapeutic target.

## Introduction

Osteosarcoma (OS) is the most common primary malignant bone tumor affecting children and young adults [1]. Approximately 80% of OS patients have metastatic disease at the time of diagnosis, presenting a major challenge for clinical management [2]. The 5-year overall survival is approximately 65%; the best predictor of long-term survival is the absence of metastatic disease at diagnosis [2]. Surgical resection with subsequent radio- and chemotherapy can dramatically improve the clinical outcome of OS patients; however, chemoresistance and pulmonary metastasis are likely to develop later on. Identifying biomarkers of metastatic progression is critical for early diagnosis and timely treatment, which could improve the prognosis of OS.

The cytoprotective chaperone protein clusterin (CLU) is involved in multiple physiological processes associated with carcinogenesis and tumor growth, including apoptotic cell death, cell cycle regulation, DNA repair, cell adhesion, tissue remodeling, lipid transport, membrane recycling, and immune system regulation [3,4]. As the major isoform, secretory apolipoprotein J (s)CLU has been extensively investigated in the context of tumor diagnosis and prognosis owing to its anti-apoptotic function. Most clinical studies have found that elevated sCLU expression is associated with tumor relapse and metastasis and is an indicator of poor prognosis [5–7]. However, others have reported contradictory or insignificant results, even for the same type of malignancy [8,9].

sCLU protein level is normally increased in cancer cells by chemo- and radiotherapy, and contributes to cancer cell resistance *in vitro* and in animal models by inhibiting apoptosis [10]. Cytoplasmic sCLU is associated with chemoresistance and is detected in a variety of advanced cancers in humans. Small interfering (si)RNA-mediated silencing of sCLU was shown to sensitize cancer cells to chemotherapy drugs [11], ionizing radiation [12], oxidative stress [13], and apoptosis induced by tumor necrosis factor-related apoptosis-inducing ligand [14]. Additionally, sCLU plays a critical role in cancer metastasis [15]; a positive correlation between sCLU level and Gleason pattern has been observed in prostate cancer, and sCLU overexpression promoted the invasion and metastasis of renal cancer and hepatocellular carcinoma [16,17]. One study demonstrated that sCLU enhanced the invasiveness and metastatic potential of prostate cancer by inducing epithelial-to-mesenchymal transition [11], suggesting that suppressing sCLU could prevent the metastatic progression of prostate cancer.

To evaluate this possibility in the context of OS, the present study investigated the relationship between sCLU expression and prognosis and chemoresistance in OS patients, and evaluated the diagnostic and predictive value of sCLU expression. We also examined the role of sCLU in OS progression *in vitro* and *in vivo* by siRNA- or short hairpin (sh)RNA-mediated gene silencing. Our results provide evidence that sCLU is a biomarker for metastatic OS as well as a potential therapeutic target for OS treatment.

## Materials and methods

### Patients

The study was approved by the institutional ethics review boards of the Department of Spine Surgery, The 107 Hospital of the People’s Liberation Army, Yantai, China. All patients provided written, informed consent prior to enrollment. Cancer tissue specimens were obtained between 1997 and 2014 from 106 OS patients who had not received any treatment prior to the study. The characteristics of the study population are shown in Table 1. Clinical staging was based on the Union for International Cancer Control Tumor Node Metastasis Classification of Malignant Tumors (Sixth Edition).

**Table 1.** The relationship between sCLU expression and clinicopathological features of osteosarcoma.

		sCLU expression				
Characteristics	No(%)	Positive	Negative	p	Survival(%)	Univariate p
**Age (years)**				0.547		0.673
≤40	64(60%)	27(42%)	37(58%)		49%	
>40	42(40%)	19(45%)	23(55%)		51%	
**Sex**				0.346		0.804
Male	70(66%)	31(44.3%)	39(55.7%)		53%	
Female	36(34%)	15(41.7%)	21(58.3%)		47%	
**Site of tumor**				1.00		0.793
Femur	56(52.8%)	20(35.7%)	36(%)		33%	
Tibia	17(16%)	10(58.8%)	7(41.2%)		27%	
Humerus	12(11.3%)	6(50%)	6(50%)		15%	
Radius,Ulna,Scapula,Rib	21(19.8%)	10(47.6%)	11(52.4%)		25%	
**Metastasis**				0.027		0.023
Yes	72(68%)	33(45.8%)	39(54.2%)		68%	
No	34(32%)	13(38%)	21(62%)		32%	
**Recurrence**				0.083		0.175
Yes	65(61%)	27(41.5%)	38(58.5%)		57%	
No	41(39%)	19(46.3%)	22(53.7%)		43%	
**Response to preoperative chemotherapy**				0.002		0.268
Good	21(20%)	6(28.6%)	15(71.4%)		25%	
Poor	52(49%)	30(57.7%)	22(42.3%)		37%	
N/A	33(31%)	10(30%)	23(70%)		38%	
**sCLU expression**						0.015
Positive		46(43.4%)	/		67.3%	
Negative		/	60(56.6%)		32.7%	

### Immunohistochemistry

Unstained tissue microarray sections (5 μm in thickness) mounted on slides were deparaffinized and hydrated in xylene and graded ethanol solutions for antigen retrieval. The slides were blocked with 3% H_2_ O_2_, goat serum, avidin solution, and biotin solution, and then incubated overnight at 4°C with anti-sCLU primary antibody (1:100 dilution in 1% bovine serum albumin) and probed with biotinylated goat anti-rabbit secondary antibody (Vector Laboratories, Burlingame, CA, USA) and high-sensitivity streptavidin-horseradish peroxidase conjugate. sCLU expression was scored as negative (−) or positive (+) when immunoreactivity was observed in < 10% and > 10% of tumor cells, respectively [18].

### Cell lines

KHOS human OS cells were purchased from the American Type Culture Collection (Shanghai, China) and grown in Roswell Park Memorial Institute 1640 medium supplemented with 10% fetal bovine serum (FBS), 25 U/ml penicillin, and 25 μg/ml of penicillin-streptomycin (P/S) at 37°C in a humidified atmosphere of 5% CO_2_.

### Transient siRNA transfection

sCLU and control siRNA were purchased from Santa Cruz Biotechnology (Shanghai, China; sc-43688). KHOS cells were transfected with the siRNAs at a final concentration of 100 nM using Lipofectamine 2000 reagent (Invitrogen, Carlsbad, CA, USA) according to the manufacturer’s instructions. After 24–72 h, total protein was extracted to assess transfection efficiency by western blot analysis.

### Stable shRNA transfection

KHOS cells were seeded in regular medium and grown to 60%–70% confluence. The medium was replaced one lacking serum and P/S and the cells were transfected with 5 μg of sCLU or control (pLKO.1 empty vector) shRNA (Thermo Fisher Scientific, Waltham, MA, USA) using Lipofectamine LTX with PLUS (Invitrogen) as recommended by the manufacturer. At 24 h post transfection, the cells were seeded in 15-cm plates and cultured in regular medium. Puromycin (1 μg/ml) was added and cells were cultured until colonies were selected and assayed for sCLU expression. Colonies with low expression of sCLU were used for subsequent experiments.

### Anti-tumor drug treatment assay

KHOS cells were seeded in 24-well plates at 5 × 10^4^ cells/well. After 24 h, the cells were transfected with sCLU or control siRNA for 24 h, then treated with 0, 1, 10, and 20 μg/ml gemcitabine (Gem) for 48 h before cell viability and apoptosis were analyzed.

### Cell viability

KHOS cell viability in response to drug treatment was evaluated with the 3-(4,5-dimethylthiazol-2-yl)-2,5-diphenyltetrazolium bromide (MTT) assay. Five replicates were prepared for each concentration tested in serial dilutions for a total of 10 concentrations with a 50% reduction in drug concentration per dilution for each multi-well plate. A 15-μl volume of MTT reagent was added to each drug-treated well, followed by incubation for 3 h at 37°C. Detergent was added to each drug-treated well and the plate was incubated overnight at room temperature while protected from light. Absorbance at 570 nm was measured using a microplate reader (Tecan Group, Mannedorf, Switzerland).

### Colony formation assay

A total of 500 cells transfected with sCLU or control shRNA were seeded in 6-well plates and cultured for 10 days at 37°C and 5% CO_2_. The number of colonies comprising > 50 cells were counted using GeneTools image analysis software (Syngene, Frederick, MD, USA) after staining the colonies with crystal violet.

### Cell invasion assay

The CultreCoat 96-well BME Cell Invasion Assay kit (R&D Systems, Shanghai, China) was used according to the manufacturer’s protocol to evaluate cell invasion. Briefly, sCLU or control siRNA-transfected cells were resuspended in fresh culture medium and transferred to the chemoinvasion chamber containing a polycarbonate filter coated with Matrigel (Chemicon International, Temecula, CA, USA) for 24 h. In the upper chamber, 3 × 10^4^ cells were seeded in FBS-free culture medium while culture medium containing 10% FBS as a chemoattractant was added to the lower chamber. The cells were allowed to migrate for 24 h, after which the chamber was washed with phosphate-buffered saline (PBS) and the cells were visualized as recommended by the manufacturer. To quantify migratory cells, the invasion chamber was dipped in 10% acetic acid, and the absorbance of the resultant solution was measured with a microplate reader at 540 nm.

### Western blot analysis

Cells were washed with ice-cold PBS and whole cell extracts were prepared in cell lysis buffer composed of 20 mmol/l Tris-HCl (pH 7.5), 0.1% Triton X-100, 0.5% deoxycholate, 1 mmol/l phenylmethylsulfonyl fluoride, 10 µg/ml aprotinin, and 10 µg/ml leupeptin. After clearing the lysate by centrifugation at 12,000 × *g* and 4°C, total protein concentration was measured with the bicinchoninic acid assay kit (Sigma-Aldrich, St Louis, MO, USE) using bovine serum albumin as a standard. Cell lysates containing 30 µg total protein were analyzed by immunoblotting. Anti-sCLU and -actin antibodies were obtained from Santa Cruz Biotechnology. Protein bands were detected by chemiluminescence using a commercial kit (Upstate, Lake Placid, NY, USA) according to the manufacturer’s instructions. The sCLU signal was quantified using ImagePro Plus v.4.0 software (Media Cybernetics, Rockville, MD, USA) and normalized to that of actin.

### Analysis of tumor development

To monitor cancer cell growth *in vivo*, cells stably transfected with sCLU or control shRNA were grown to 80% confluence, harvested by incubation with trypsin-EDTA, washed twice with PBS, and resuspended to a final concentration of 2.5 × 10^6^ cells/ml. A 100-µl volume of the cell suspension was injected into the back (right side) of 4-week-old male nude mice (n = 10 per group). Tumors were allowed to develop for 5 weeks (50–100 mm^3^) before the mice were treated with Gem (100 mg/kg) via intraperitoneal injection (two times/week). Tumor growth was monitored weekly by bioluminescence imaging using the IVIS Imaging system (Caliper Life Sciences, Waltham, MA, USA). At the end of 5 weeks, the animals were sacrificed and the tumors were removed and processed for histological analysis.

It was previously reported that injection of 1 × 10^5^ SGC-7901, 1 × 10^5^ U-2OS/NP, or 1 × 10^5^ SJSA-1 cells injected into the vein of nude mice resulted in lung metastasis [19–21]; or 2 × 10^5^ breast cancer or 5 × 10^5^ human mammary epithelial cells [22,23] into the vein of nude mice resulted in lung metastasis. In our study, lung metastasis was experimentally induced by intra-tibial injection of 3 × 10^5^ cells for 3 weeks.

### *Apoptosis detection* in vivo

Tumors harvested from mice were cut into 5-μm sections and apoptotic cells were detected with a terminal deoxynucleotidyl transferase nick-end labeling (TUNEL) kit (Chemicon, Temecula, CA, USA) according to the manufacturer’s protocol. Briefly, the sections were deparaffinized and rehydrated; endogenous peroxidase was blocked and antigen retrieval was performed. The TUNEL reaction mixture (50 μl) was added to the sections, followed by incubation in the dark for 60 min at 37°C in a humidified atmosphere. After washing with PBS, the sections were mounted with Fluoromount mounting medium (Sigma Aldrich) and examined with a fluorescence microscope (Olympus, Melville, NY, USA).

### Statistical analysis

Differences in immunohistochemistry data were analyzed with the Kruskal-Wallis and Mann-Whitney U tests for comparisons involving three and two groups, respectively. The relationship between sCLU expression and each clinicopathological parameter was analyzed with the Mann-Whitney U test. Receiver operating characteristic (ROC) curves were generated and the area under the curve was calculated with a 95% confidence interval. ROC curve analysis was performed for sCLU expression. Survival curves were obtained by the Kaplan–Meier method and survival was analyzed with the log-rank test. A univariate logistic regression analysis was performed for these correlations to determine the significance of the observed features. All *in vitro* experiments were performed in triplicate and were repeated at least three times, and *in vivo* experiments were performed at least twice. Values are expressed as mean ± SEM. Differences between group means were assessed with the Student’s t test; P < 0.05 was considered significant.

## Results

### sCLU is overexpressed in OS tissue

**Figure 1.**
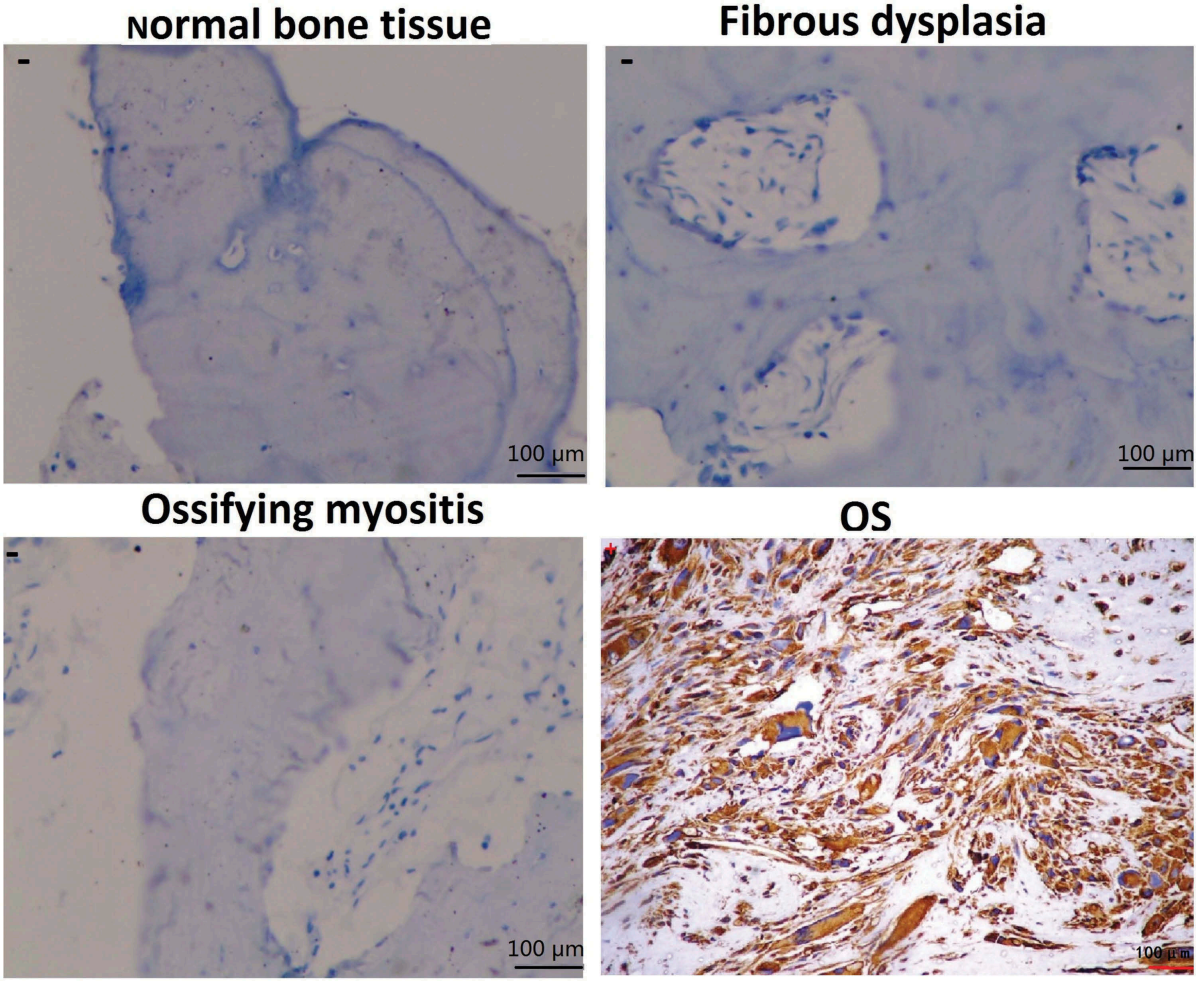
sCLU immunoreactivity in normal bone, fibrous dysplasia, ossifying myositis, and OS tissues. The ‘−’ and ‘+’ symbols in the cytoplasm of cells indicate negative and positive staining, respectively (200× magnification).

**Figure 2.**
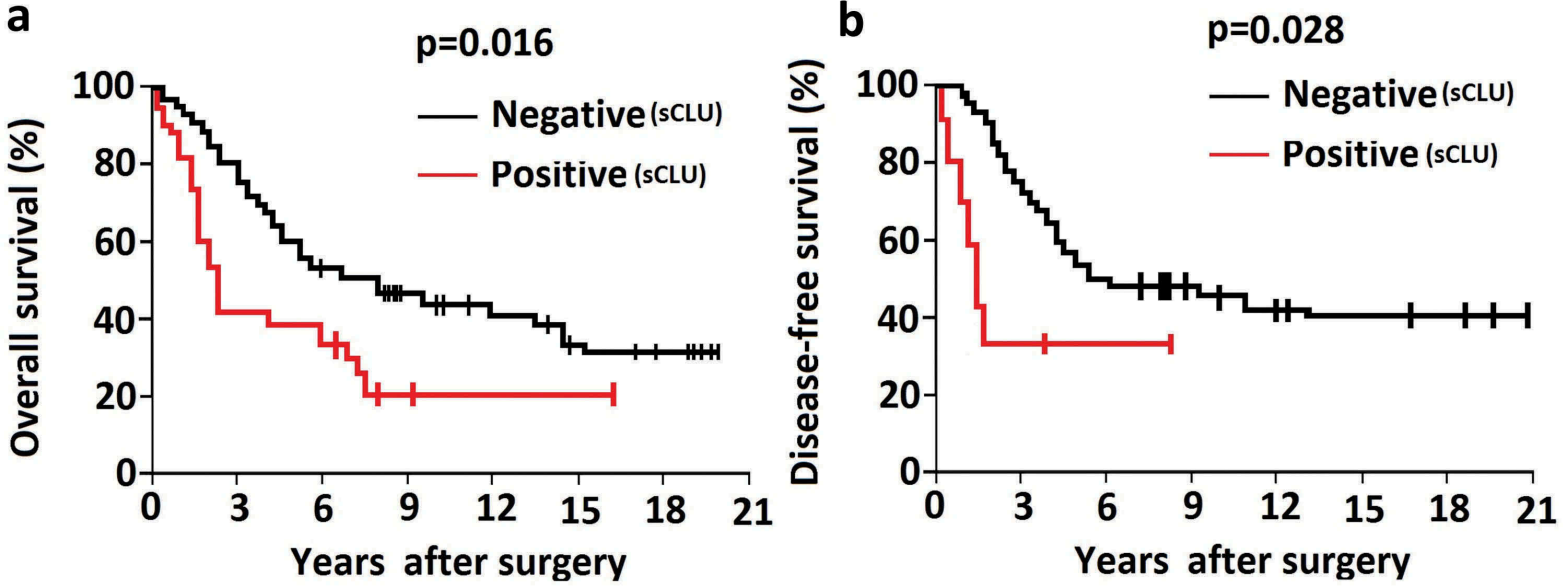
Association between sCLU protein expression and clinical outcome in OS patients. A, B, Kaplan–Meier curves depicting overall survival (a) and disease-free survival (b) rates in OS patients positive and negative for sCLU expression.

Immunohistochemical analysis of clinical specimens revealed that normal bone, fibrous dysplasia, and ossifying myositis tissues were negative for sCLU expression (Figure 1). In contrast, 46 of the 106 OS patients (43.4%) were positive for sCLU cytoplasmic staining while 60 (56.6%) were negative (Figure 2).

### High sCLU expression is associated with poor outcome in OS patients

The correlations between sCLU expression and clinicopathologic characteristics of the study population are shown in Table 1. In the 106 patients, sCLU expression was significantly associated with post-operative clinical outcome at the median 78.6-month follow-up evaluation. Furthermore, sCLU immunopositivity was associated with metastasis at initial diagnosis (P = 0.023; Table 1) and poorer overall and disease-free survival (Figure. 2(a,b)), and was correlated with poor histological response after neoadjuvant chemotherapy (Table 1). sCLU *silencing increases apoptosis and decreases proliferation and invasion of KHOS cells*

**Figure 3.**
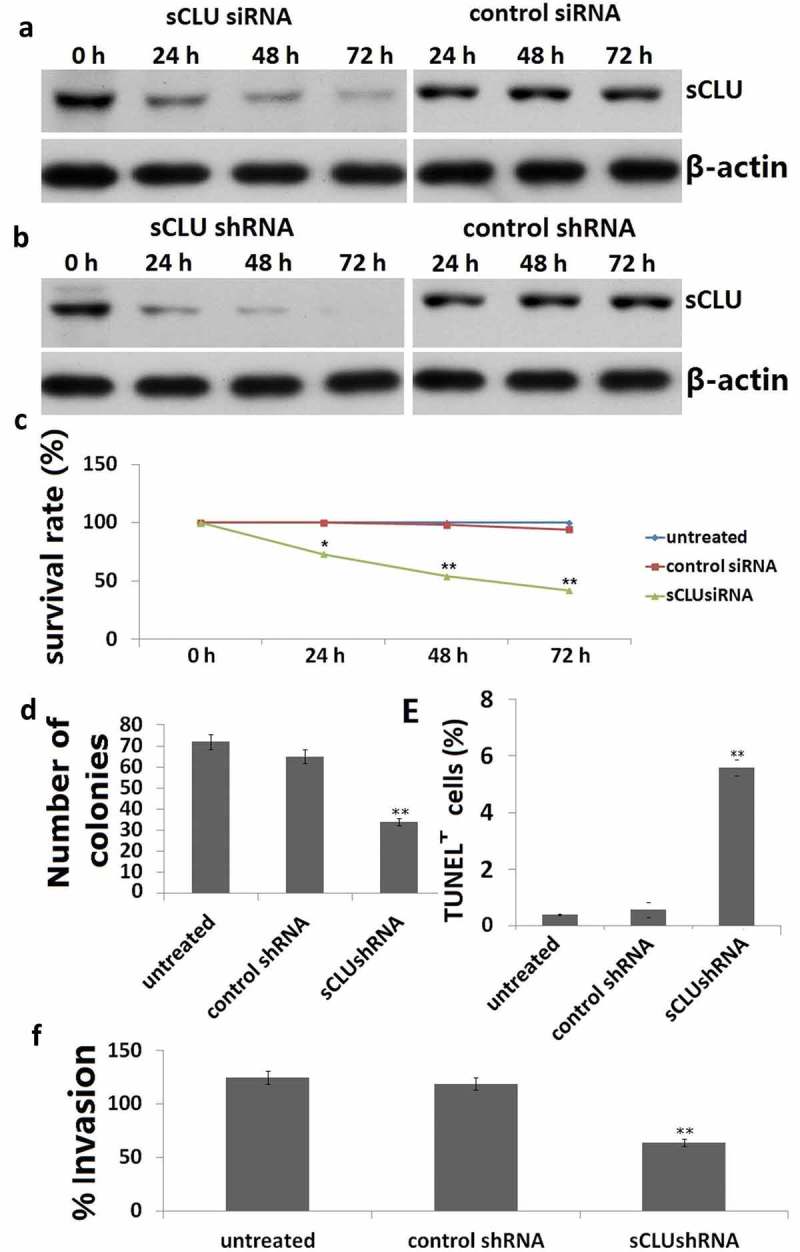
Effect of sCLU silencing on apoptosis, viability, proliferation, and invasion of KHOS cells *in vitro*. (a), Cells were transfected with sCLU siRNA for 24–72 h, and sCLU protein expression was detected by western blotting. (b), sCLU protein expression in KHOS cells stably transfected with sCLU shRNA was detected by western blotting. (c), Effects of sCLU or control siRNA transfection on the viability of KHOS cells. **P < 0.05, **P < 0.01. (d), Representative results of the colony formation assay for KHOS cells transfected with control or sCLU shRNA. **P < 0.01. (e), Effects of sCLU or control siRNA transfection on KHOS cell apoptosis. **P < 0.01. (f), Evaluation of KHOS cell invasion with the transwell assay following sCLU knockdown. **P < 0.01. Scale bar, 50 μm. Results are representative of three independent experiments.

A western blot analysis revealed that sCLU protein was highly expressed in KHOS cells (Figure 3(a)). We transfected KHOS cells with *sCLU* siRNA and found that the knockdown efficiency as determined by western blotting increased in a time-dependent manner, with 33%, 65%, and 75% depletion observed 24, 48, and 72 h post transfection, respectively (Figure 3(a)). As expected, the control siRNA had no effect on sCLU protein expression (Figure 3(a)). sCLU expression was also reduced KHOS cells stably transfected with *sCLU* shRNA (Figure 3(b)).

We next examined the effect of *sCLU* silencing on the viability, apoptosis, and invasion of KHOS cells at 24, 48, and 72 h after transfection for 48 h. *sCLU* knockdown decreased the viability of KHOS cells in a time-dependent manner, as determined with the MTT assay (Figure 3(c)). The results of the colony formation assay for *in vitro* tumorigenesis revealed that the rate of colony formation was lower in cells transfected with *sCLU* shRNA (35 ± 8) as compared to those transfected with control shRNA (67 ± 10) or without transfection (69 ± 10) (Figure 3(d)). *sCLU* knockdown increased the rate of apoptosis relative to control and non-transfected cells (25.7% ± 5.3% vs. 3.6% ± 0.7% vs. 4.2% ± 0.8%, P < 0.01; Figure 3(e)), with a corresponding decrease in the number of invading cells (67 ± 9 vs. 128 ± 12 vs. 136 ± 16, P < 0.01; Figure 3(f)).

### *sCLU* silencing sensitizes KHOS cells to Gem by inducing apoptosis

**Figure 4.**
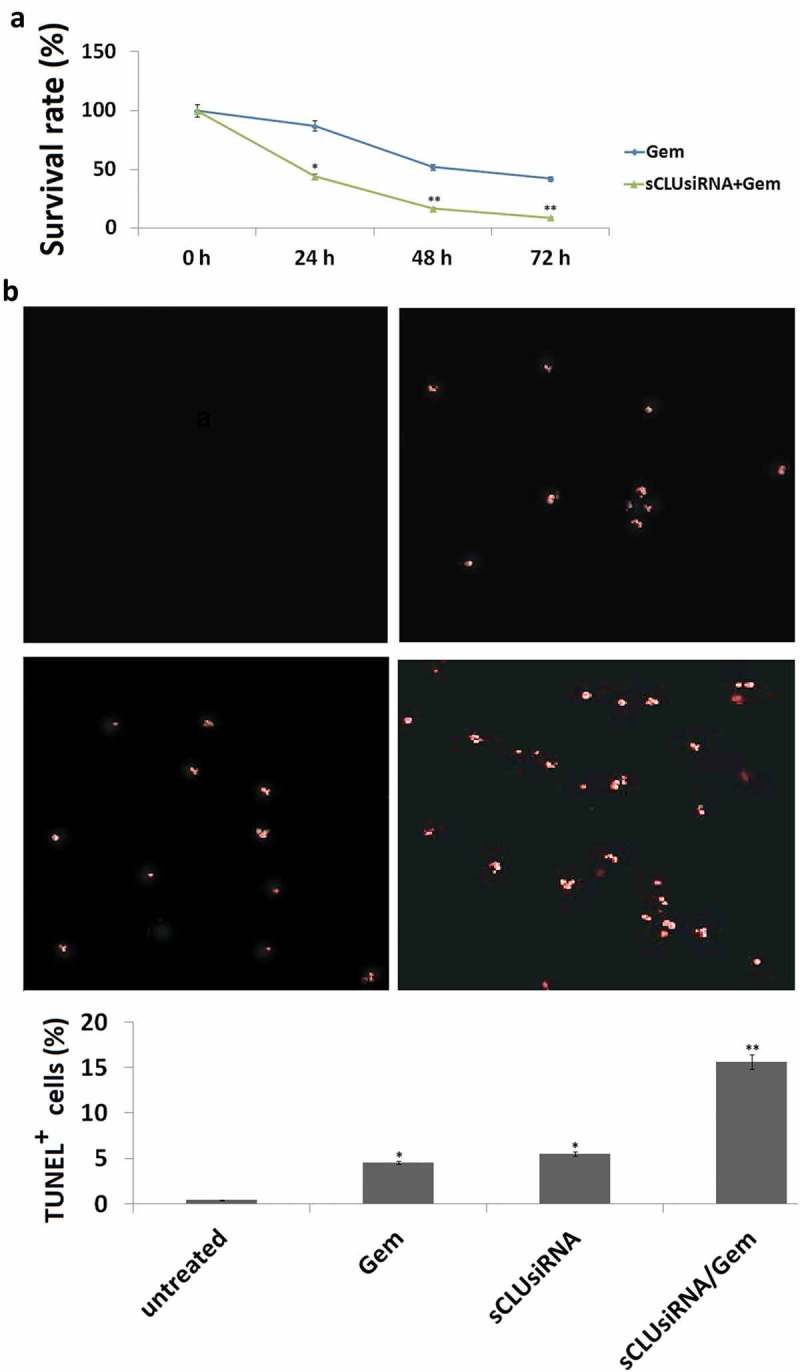
Effect of sCLU knockdown combined with Gem treatment on apoptosis and viability of KHOS cells. (a), KHOS cells were transfected with sCLU or control siRNA with or without Gem treatment (1–20 μg/ml) for 48 h; cell viability was detected with the MTT assay. (b), KHOS cells were transfected with sCLU or control siRNA with or without Gem treatment (10 μg/ml) for 48 h; cell apoptosis was detected with the TUNEL assay. *P < 0.05, **P < 0.01.

To test the hypothesis that inhibiting sCLU can increase the sensitivity of KHOS cells to Gem-mediated apoptosis, cells were transfected with *sCLU* or control siRNA and then treated with Gem. sCLU-depleted cells showed increased sensitivity to Gem (0, 1, 10, and 20 μg/ml), as evidenced by the lower rate of proliferation in the MTT assay (Figure 4(a)) and a higher rate of apoptosis in the TUNEL assay (10 μg/ml) (Figure 4(b)). In contrast, control siRNA transfection had no effect on Gem-induced cell apoptosis or growth inhibition.

### *sCLU* silencing reduces tumor growth and lung metastasis in vivo

**Figure 5.**
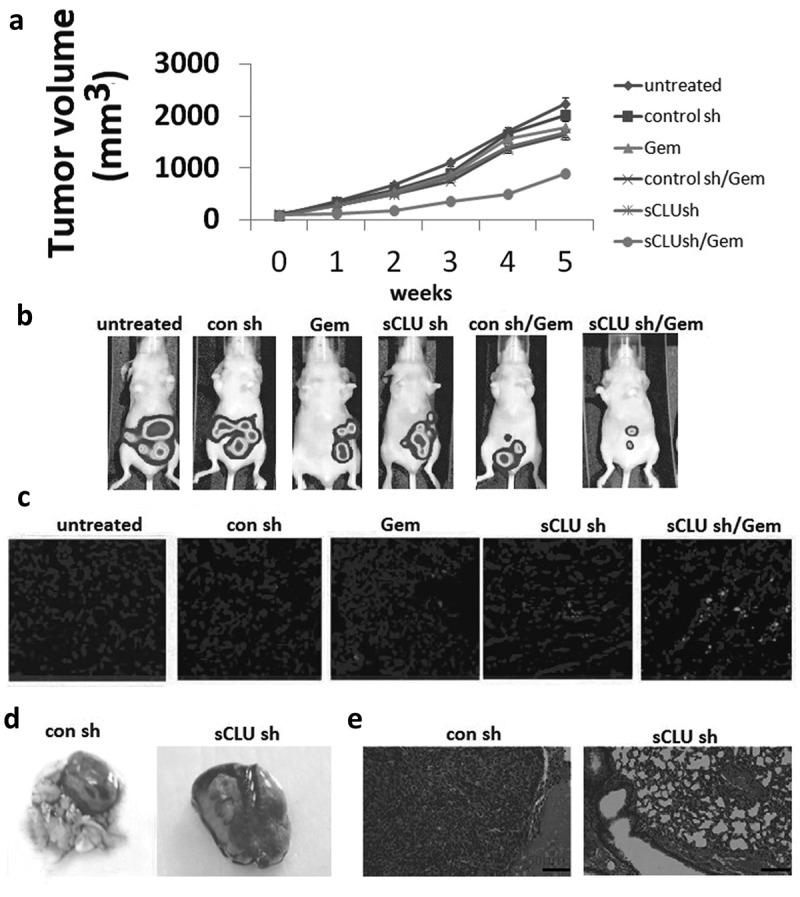
Combined effect of sCLU silencing and Gem treatment in a mouse xenograft model. (a), Stable sCLU or control shRNA clones were implanted into nu/nu mice (n = 5); when the tumor diameter was 0.06–0.1 cm^3^, the mice were treated with Gem. Tumor volume was calculated as described in Methods. *Combined P = 0.026 vs. Gem or sCLU shRNA. (b), Bioluminescence images of tumors in mice implanted with stable sCLU or control shRNA clones and treated with Gem at 5 weeks post-treatment. (c), TUNEL staining of tumor tissues. (d), Effect of sCLU knockdown on the formation of metastatic lesions in an orthotopic lung mouse xenograft model. Representative gross images of lungs show multiple massive tumors (arrows) in a mouse from the control shRNA group, in contrast to the minuscule tumor in a mouse from the sCLU shRNA group. (e), Micrographs of hematoxylin and eosin-stained tumors in control and sCLU shRNA group mice. Scale bar, 50 μm.

To validate the above findings *in vivo*, KHOS cells stably expressing *sCLU* or control shRNA were injected into the back of mice and tumor growth was monitored via bioluminescence imaging. After 3–4 weeks when the mean tumor size was 60–100 mm^3^, the mice were randomized into the following six treatment groups (n = 5 each): untreated, Gem, sCLU shRNA, Gem + sCLU shRNA, control shRNA, and control shRNA + Gem. The treatments were continued for 5 weeks and tumor burden was monitored weekly by bioluminescence imaging. sCLU shRNA or Gem alone had little effect on the time course of tumor development, but their combination markedly reduced tumor growth; no tumors were detected in these mice after 5 weeks (Figure 5(a)). In contrast, control shRNA transfection had no effect on tumor growth (Figure 5(a,b)). The results of the TUNEL assay indicated that the apoptotic fraction was increased in the combined treatment group relative to control mice (Figure 5(c)). Mice injected with sCLU-depleted cells remained tumor-free after 5 weeks, whereas control mice showed lung metastases (Figure 5(d,e)). Collectively, these results demonstrate that sCLU promotes tumor growth and lung metastasis.

## Discussion

This study had three novel findings. We found that sCLU is overexpressed in human OS tissues and that this is correlated with metastasis, poor prognosis, and poor response to neoadjuvant chemotherapy. Finally, *sCLU* silencing reduced the oncogenic and metastatic potential of KHOS cells and increased their sensitivity to the chemotherapeutic drug Gem. These results suggest that sCLU is a promising therapeutic target for OS treatment.

sCLU is a small stress-induced cytoprotective chaperone protein that plays an important role in cell proliferation, multidrug resistance, metastasis, and tumor progression [24]. Aberrant sCLU expression in the serum or tumor tissues of patients with primary cancers is considered as a useful biomarker for disease diagnosis and surveillance [8,25–27]. In our study, we observed that sCLU was overexpressed in OS tissue relative to non-tumor or non-metastatic tumor tissue, implying that sCLU contributes to OS progression by acting as an oncogene. In addition, elevated sCLU expression was associated with shorter disease-free and overall survival in OS patients, and sCLU expression was an independent prognostic factor for these patients. We also observed that high sCLU expression is independent of metastatic disease status at diagnosis. Thus, sCLU may be a useful diagnostic and prognostic biomarker for OS as well as a therapeutic target for its treatment.

sCLU expression was previously shown to be a useful marker for predicting response to preoperative chemotherapy and clinical outcome in patients with locally advanced breast cancer [28]. sCLU overexpression stabilized the B cell lymphoma 2-associated X protein (Bax)-Ku70 complex, inhibited Bax-dependent apoptosis, and increased resistance to chemoradiation in patients with locally advanced rectal cancer [29]. In our study, we found that high levels of sCLU were correlated with chemoresistance in OS, suggesting that sCLU confers drug resistance to tumor cells and that therapeutic strategies that target sCLU may sensitize these cells to the effects of chemotherapy.

Knocking down *sCLU* expression by siRNA transfection or lentiviral transduction of shRNA inhibited breast and ovarian cancer cell proliferation, migration, and invasion and caused cell cycle arrest [30,31]. Here we used gene silencing technology to assess the clinical value of *sCLU* depletion in KHOS cells and found that it reduced their growth, survival, and clonogenicity and increased their sensitivity to Gem. In addition, targeted knockdown of *sCLU* suppressed cell invasion *in vitro* and tumor growth and lung metastasis *in vivo*, which is consistent with previous reports [17].

Cancer progression and recurrence following chemotherapy have been attributed to inherent or acquired resistance to chemotherapy drugs. Neoadjuvant chemotherapy was shown to activate sCLU, leading to the development of paclitaxel chemoresistance [32]. On the other hand, *sCLU* knockdown sensitized breast cancer cells to neoadjuvant chemotherapy-induced apoptosis [33]. A similar finding was reported in lung cancer cells [34]. Thus, targeting sCLU could be a general strategy for increasing tumor cell sensitivity to chemotherapy and improving cancer treatment outcome.

## Conclusion

The results of this study suggest that sCLU overexpression is correlated with metastasis, poor prognosis, and resistance to neoadjuvant chemotherapy in patients with OS. Silencing sCLU inhibited cell invasion and increased the sensitivity of OS cells to Gem treatment. Our findings indicate that targeting sCLU could improve the prognosis of OS patients with elevated sCLU expression.
